# New findings in the roles of Cyclin-dependent Kinase inhibitors 2B Antisense RNA 1 (*CDKN2B-AS1*) rs1333049 G/C and rs4977574 A/G variants on the risk to coronary heart disease

**DOI:** 10.1080/21655979.2020.1827892

**Published:** 2020-10-14

**Authors:** Wei Yuan, Wei Zhang, Wei Zhang, Zhong-Bao Ruan, Li Zhu, Yu Liu, Yuan-Yuan Mi, Li-Feng Zhang

**Affiliations:** a Department of Cardiology, Taizhou People’s Hospital, Taizhou, China; b Department of Infectious Disease, Taizhou People’s Hospital, Taizhou, China; c Department of Oncology, Taizhou People’s Hospital, Taizhou, China; d Department of Cardiology, The Affiliated Changzhou No.2 People’s Hospital of Nanjing Medical University, Changzhou, China; e Department of Urology, Affiliated Hospital of Jiangnan University, Wuxi, China; f Department of Urology, The Affiliated Changzhou No.2 People’s Hospital of Nanjing Medical University, Changzhou, China

**Keywords:** Coronary heart disease, *CDKN2B-AS1*, Genetic variation, Variant, Analysis

## Abstract

The relationship between Cyclin-Dependent Kinase Inhibitors 2B Antisense RNA 1 (*CDKN2B-AS1*) variants rs1333049 G/C and rs4977574 A/G and the risk of coronary heart disease is unclear. We conducted an update analysis incorporating odds ratios and 95% confidence intervals to assess the correlation. Furthermore, we used *in silico* analysis to investigate the genes and proteins that interact with CDKN2B. Fifty case-control studies with a sample size of 35,915 cases and 48,873 controls were involved. We revealed that the rs1333049 C allele could increase the risk of coronary heart disease in the overall analysis (allele comparison, OR = 1.13, 95%CI = 1.05–1.21, *P* = 0.001; homozygous contrast, OR = 1.29, 95%CI = 1.11–1.49, *P* = 0.001; dominant comparison, OR = 1.14, 95%CI = 1.03–1.27, *P* = 0.011; recessive comparison, OR = 1.21, 95%CI = 1.10–1.34, *P* < 0.001). In subgroup analysis, positive correlations were detected in studies involving West and East Asians and in population-based control studies. The rs4977574 G allele was also a risk factor for coronary heart disease (allelic comparison, *P* = 0.001; heterozygous comparison, *P* = 0.003; homozygous comparison, *P* < 0.001; dominant comparison, *P* = 0.001). These results indicate correlation of *CDKN2B-AS1* rs1333049 G/C and rs4977574 A/G variants may be correlated with the risk of coronary heart disease.

**Abbreviations** CDK: Cyclin Dependent Kinase; CCND: G1/S-specific cyclin-D; CDKN: Cyclin Dependent Kinase Inhibitor; GWAS: Genome-wide association study; CDKN2B-AS1: Cyclin-Dependent Kinase Inhibitors 2B Antisense RNA 1; CHD: Coronary heart disease; MAF: minor allele frequencies; HWE: Hardy-Weinberg equilibrium of controls; CI: confidence interval; COL8A2: Collagen type VIII alpha 2 chain; HB: Hospital-based; ORs: odds ratios; ITGA11: Integrin subunit alpha 11; LTBP: Latent transforming factor beta binding protein; PB: Population-based; IBC: Itmat Broad Care; NA: Not applicable; PCR-RFLP: polymerase chain reaction-restriction fragment length polymorphism; MI: Myocardial Infarction; SNP: single nucleotide polymorphism; SMAD: Mothers against decapentaplegic homolog; RT-PCR: Real-time polymerase chain reaction; UK: United Kingdom

## Graphical abstract

**Figure:**
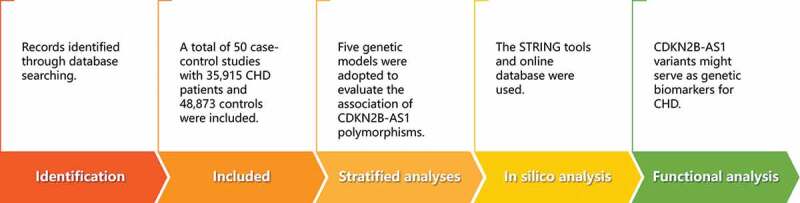

## Introduction

Coronary heart disease (CHD) is characterized by coronary artery stenosis and leading to occlusion. This disease is one of the leading causes of disability and death globally [1]. The exact pathogenesis of CHD is unclear; however, evidence indicate a crucial role of genetic factors in the development of CHD [2]. Genome-wide association studies have provided evidence of a correlation between common variations on specific chromosome location 9p21.3 and susceptibility to cardiovascular diseases including atherosclerosis-related ischemia and coronary heart disease [3,4].

Cyclin-Dependent Kinase Inhibitors 2B Antisense RNA 1 (*CDKN2B-AS1*) also known as Antisense Noncoding RNA in the INK4 locus (ANRIL) is a potential CHD candidate gene located within the CDKN2A-CDKN2B gene cluster on human chromosome 9 (9p21.3). *CDKN2B-AS1* can also encode a large antisense non-coding RNA, and prior studies have suggested the role of *CDKN2B-AS1* gene in the progression of CHD by regulating the expression of *CDKN2B* and other genes in cardiac tissue [5]. Inhibition of *CDKN2B-AS1* in vascular smooth muscle could affect the expression of extra-cellular matrix remodeling genes, indicating a pivotal role in vascular function [6]. Abnormal *CDKN2B-AS1* expression in atherosclerotic lesions can promote atherosclerosis and thrombosis [7,8]. Therefore, it is plausible that variants in the *CDKN2B-AS1* gene are associated with atherosclerosis-related diseases, including CHD.

Polymorphisms of *CDKN2B-AS1* have been investigated previously and have been correlated with susceptibility to various diseases that include ischemic stroke, glaucoma, gout, and cancer [9–12]. Prior studies have assessed the potential association between *CDKN2B-AS1* variants and the likelihood of CHD. The variant rs4977574 (A/G) is considered as a non-protein-coding variation located on chromosome 9p21.3 adjacent to *Cyclin-Dependent Kinase Inhibitor 2B* (*CDKN2B*). Up to now, the A to G variation can be correlated with early onset of CHD. This variation affects the expression level of *CDKN2B* in many tissues including coronary artery smooth muscle cells [5,13]. For rs1333049, the carrying of C allele was found to be a risk factor for CHD patients in West Siberia. The SNP (single nucleotide polymorphism) allele C, when present in the heterozygous genotype (GC) elevated CHD risk by 15–20% and when present in the homozygous SNP genotype (CC) elevated CHD risk by 30–40% [14,15]. Most of these studies are pilot researches, and their findings are far from conclusive [16,17]. In 2018, two meta-analyses explored the association between *CDKN2B-AS1* polymorphisms and coronary artery disease. One analysis involved only 9 studies based on the rs1333049 variant [18] and the other included 6 studies involving the rs4977574 polymorphism [19]. Up to now, there is still no prior study to determine whether *CDKN2B-AS1* rs1333049 C and rs4977574 G allele can be used as a marker for the diagnosis or prognosis of CHD. The aim of the present research was to identify all eligible case-control studies to comprehensively investigate the correlation of *CDKN2B-AS1* polymorphisms and CHD [20–58]. Furthermore, we used *in silico* analysis to investigate the genes and proteins that interact with *CDKN2B.*

## Materials and methods

### Search strategy

A literature search of Embase, PMC, Google Scholar, and Chinese Wanfang databases for relevant published articles was performed using the search term (‘rs4977574’ OR ‘rs1333049’ OR “CDKN2B antisense RNA” OR “CDKN2B-AS” OR “9p21” OR “ANRIL”) AND (“variant” OR “variant” OR “SNP”) AND (“myocardial infarction” OR ”coronary artery disease’). The most recent search update was 1 June 2020. Besides the use of databases, eligible studies were also retrieved by searching the references cited in the published articles.

### Inclusion criteria and exclusion criteria

A publication was included in the analysis only if it met the following criteria: (a) Case–control study addressing the relationship between *CDKN2B-AS1* rs1333049 and rs4977574 variants and CHD; (b) Study providing available genotypic frequencies of 9p21 region polymorphisms; and (c) Full text in English or other languages. Major exclusion criteria were (a) Duplicated studies using the same data; (b) Absence of a control group; and (c) No relevant to *CDKN2B-AS1* variants and CHD.

### Data extraction

Information retrieved from the included studies was as follows: First author name, date of publication, region, and ethnicity of populations used, primary outcome, source of the control samples, total sample size, gene distribution of *CDKN2B-AS1* variants, evaluation of Hardy-Weinberg equilibrium (HWE), and the genotyping method. In addition, studies including Asian population were divided into East Asia and West Asia. Two investigators independently carried out data extraction and quality evaluation and differences between them were resolved by discussions until a consensus was reached.

### Statistical analyses

Strength of the correlation between *CDKN2B-AS1* rs1333049 and rs4977574 variants and CHD susceptibility was investigated using odds ratios (ORs) together with 95% confidence intervals. Five genetic models were adopted to assess the likelihood of *CDKN2B-AS1* polymorphisms. For SNP rs1333049 G/C, the allele comparison represents C-allele versus (vs.) G-allele; heterozygous contrast refers to CG vs. GG; homozygous contrast represents CC vs. GG; dominant model represents CC + CG vs. GG; and recessive model refers to CC vs. CG + GG. For SNP rs4977574 A/G, the five genetic models were G-allele vs. A-allele, GA vs. AA, GG vs. AA, GG+GA vs. AA, and GG vs. GA + AA. Cochran’s *Q* statistic was performed to calculate the heterogeneity between ORs. If the probability (*P*) value < 0.05 was considered as statistically significant, indicating heterogeneity among studies. In this case, a random-effects model was adopted. Otherwise, we carried out a fixed-effects model. The HWE *P* value was calculated using the Fisher’s exact test, with a *P* value < 0.05 indicating significant bias. Stratification analyses were carried out to investigate the strength of ethnicity, control source, and type of primary outcome. Begg’s funnel plot was adopted to assess the potential publication bias. *P* < 0.05 represents the significance exists. Sensitivity analyses were used to test the reliability of the included studies. All statistical methods were referring to the STATA 11.0 software of StataCorp (College Station, TX).

### In silico analysis of CDKN2B

Differentially expressed genes between the CHD and control groups in the overall population were evaluated using an online database. Moreover, we checked the minor allele frequencies (MAFs) in worldwide populations based on the online database (https://www.ncbi.nlm.nih.gov/snp). The protein–protein interactions of CDKN2B were investigated using the STRING tools (https://string-db.org/cgi/input.pl).

## Results

### Characteristics of eligible studies

**Figure 1.**
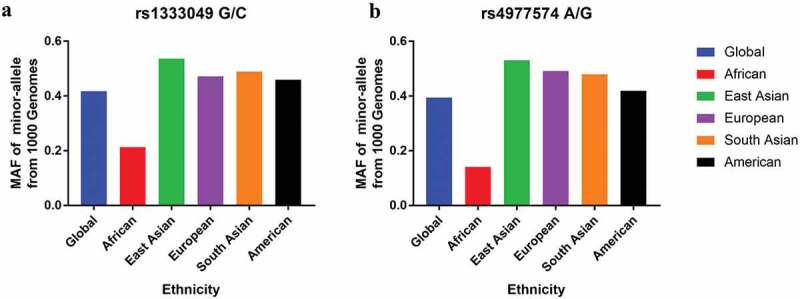
Minor allele frequencies of *CDKN2B-AS1* rs1333049 G/C and rs4977574 A/G polymorphisms in various races.

Fifty case-control studies comprising 35,915 CHD patients and 48,873 control subjects met the inclusion criteria and were summarized in the present study (Table 1). For the rs1333049 G/C variant, 33 studies with 20,365 cases and 29,413 controls were involved. In subgroup analysis by ethnicity, the sample population of 14 studies was of Europeans, 18 studies were of Asian descendants (divided into West Asians and East Asians), and one study was on the African population. Stratification analysis based on the source of controls used revealed that 14 studies were hospital based and 17 studies were population based. In a subgroup analysis by disease type, 22 studies focused on unclassified coronary artery disease and 11 studies focused on myocardial infarction. For the rs4977574 A/G polymorphism, the sample population of 8 studies was of European descendants and 9 studies was of Asian populations (4 studies were of West Asians and 5 were of East Asians). Stratification analysis based on the source of controls revealed 7 studies as hospital based and 10 studies as population based. We also determined the MAFs in the overall and sub-populations. The MAFs for the SNP rs1333049 G/C variant were as follows: global population, 0.418; Africans, 0.213; East Asians, 0.537; European descendants, 0.472; South Asians, 0.491; and Americans, 0.455. In the current study, the MAF in case was 0.521; and in control was 0.489. The MAFs for the SNP rs4977574 were as follows: global population, 0.395; Africans, 0.141; East Asians, 0.531; Europeans, 0.492; South Asians, 0.484; and Americans, 0.416 (Figure 1). In the present study, the MAF in case was 0.537; and in control was 0.483.

**Table 1.** Study characteristics of CDKN2B-AS1 rs1333049 G/C and rs4977574 A/G variants included in the present analysis.

First author	Year	Origin	Type	Ethnicity	Source of control	Case	Control	Case			Control			HWE	Method
rs1333049 G/C								CC	CG	GG	CC	CG	GG		
Suleiman	2019	Iraq	CAD	West Asian	Hospital based	50	50	9	22	19	4	23	23	0.595	Primex PCR
Shakhtshneider	2019	Russia	MI	Caucasian	Population based	118	2610	39	51	28	554	1330	726	0.228	RT-PCR
Kalpana	2019	India	CAD	West Asian	Population based	91	436	30	38	23	102	222	112	0.693	MassARRAY
Huang	2019	Mainland China	CAD	East Asian	Hospital based	501	496	110	263	128	94	254	148	0.417	MassARRAY
Kashyap	2018	India	CAD	West Asian	Hospital based	512	272	117	316	79	46	176	50	<0.001	PCR-RFLP
Yang	2018	Mainland China	CAD	East Asian	Hospital based	542	549	111	269	162	100	273	176	0.743	MassARRAY
Pignataro	2017	Italy	CAD	Caucasian	NA	711	755	251	342	118	215	391	149	0.229	NA
Li	2017	Mainland China	CAD	East Asian	NA	555	480	198	239	118	129	223	128	0.121	TaqMan
Haslacher	2016	Austria	MI	Caucasian	Population based	493	431	118	236	139	97	222	112	0.514	Taqman
Foroughmand	2015	Iran	CAD	West Asian	Hospital based	170	100	31	111	28	25	67	8	<0.001	ARMS-PCR
Cakmak	2015	Turkey	CAD	Caucasian	Hospital based	220	240	54	120	46	85	115	40	0.917	RT-PCR
Pinos	2014	Spain	CAD	Caucasian	Hospital based	152	343	45	53	54	105	153	85	0.052	TaqMan
Pinos	2014	Japan	CAD	East Asian	Hospital based	742	920	158	373	211	193	485	242	0.082	TaqMan
Jansen	2014	Norway	CAD	Caucasian	Population based	818	2094	238	368	212	647	1009	438	0.224	MassARRAY
Gong	2014	Mainland China	CAD	East Asian	Hospital based	545	725	133	248	164	160	358	207	0.824	MassARRAY
Bhanushali	2013	India	CAD	West Asian	Hospital based	97	151	33	57	7	34	80	37	0.461	Taqman
Bhanushali	2013	India	MI	West Asian	Hospital based	120	151	38	60	22	34	80	37	0.461	Taqman
Zeng	2013	Mainland China	CAD	East Asian	Population based	359	398	110	168	81	75	197	126	0.897	PCR-RFLP
Ahmed	2013	Pakistan	MI	West Asian	Hospital based	294	290	63	166	65	23	180	87	<0.001	Taqman
Qi	2012	Mainland China	MI	East Asian	Hospital based	142	192	21	79	42	43	99	50	0.651	PCR-RFLP
Lin	2011	Taiwan	MI	East Asian	Hospital based	423	1361	105	218	100	311	655	395	0.213	Taqman
Guo	2011	Mainland China	CAD	East Asian	Population based	670	1340	156	327	187	358	661	321	0.643	RT-PCR
Xie	2011	Mainland China	CAD	East Asian	Population based	2305	1061	659	1140	506	241	525	295	0.810	Taqman
Scheffold	2011	Germany	MI	Caucasian	Population based	976	999	246	518	212	205	502	292	0.688	RT-PCR
Mendonca	2011	Portugal	CAD	Caucasian	Population based	723	683	258	348	117	200	321	162	0.136	Taqman
Ghazouani	2010	Tunisia	CAD	African	Population based	292	323	72	137	83	88	151	84	0.244	Taqman
Saleheen	2010	Pakistan	MI	West Asian	Population based	2587	2573	697	1273	617	609	1290	674	0.865	IBC array
Peng	2009	Mainland China	MI	East Asian	Population based	520	560	156	265	99	116	285	159	0.572	Taqman
Hiura	2008	Japan	MI	East Asian	Population based	586	2432	170	279	137	592	1204	636	0.638	Taqman
Hinohara	2008	Korea	CAD	East Asian	Population based	679	706	186	335	158	161	353	192	0.959	Taqman
Hinohara	2008	Japan	CAD	East Asian	Population based	604	1151	178	312	114	259	606	286	0.069	Taqman
Samani	2007	German	MI	Caucasian	Population based	844	1605	158	453	233	425	831	349	0.130	GeneChip
Samani	2007	UK	CAD	Caucasian	Population based	1924	2936	586	960	378	676	1431	829	0.222	GeneChip
rs4977574 A/G								GG	GA	AA	GG	GA	AA		
Hua	2020	Mainland China	CAD	East Asian	Hospital based	598	257	152	297	149	48	122	87	0.651	MassARRAY
Temel	2019	Turkey	CAD	Caucasian	Hospital based	71	153	14	33	24	38	76	39	0.936	PCR-RFLP
Kalpana	2019	India	CAD	West Asian	Population based	90	436	31	36	23	100	230	106	0.249	MassARRAY
Tang	2017	Mainland China	CAD	East Asian	Hospital based	289	338	116	136	37	166	134	38	0.172	RT-PCR
Cao	2016	Mainland China	CAD	East Asian	Hospital based	565	541	176	272	117	134	255	152	0.191	PCR-RFLP
Matsuoka	2015	Japan	MI	East Asian	Population based	1822	2284	476	898	448	501	1132	651	0.831	Suspension array
Beigi	2015	Iran	CAD	West Asian	Hospital based	100	93	34	44	22	32	44	17	0.784	TaqMan
Huang	2014	Mainland China	CAD	East Asian	Hospital based	590	482	163	305	122	77	267	138	0.006	MassARRAY
Sakalar	2013	Turkey	MI	Caucasian	Hospital based	44	28	14	22	8	4	11	13	0.513	PCR-RFLP
Saade	2011	Lebanon	CAD	West Asian	Population based	1520	423	627	685	208	156	195	72	0.409	Illumina
Saleheen	2010	Pakistan	MI	West Asian	Population based	2584	2576	746	1242	596	636	1298	642	0.693	IBC array
Helgadottir	2007	Iceland	MI	Caucasian	Population based	2215	4806	556	1105	554	964	2335	1507	0.275	GWAS
Helgadottir	2007	Philadelphia	MI	Caucasian	Population based	569	495	180	286	103	119	246	130	0.901	GWAS
Helgadottir	2007	Atlanta	MI	Caucasian	Population based	577	1254	188	274	115	325	597	332	0.090	GWAS
Helgadottir	2007	Durham	MI	Caucasian	Population based	1132	714	316	549	267	144	383	187	0.040	GWAS
Samani	2007	German	MI	Caucasian	Population based	860	1643	169	452	239	463	826	354	0.688	GeneChip
Samani	2007	UK	CAD	Caucasian	Population based	1924	2937	605	937	382	698	1435	804	0.243	GeneChip

CAD: Coronary artery disease; HWE: *P* value for Hardy-Weinberg equilibrium in controls; GWAS: Genome-wide association study; IBC: Itmat Broad Care; MI: Myocardial Infarction; NA: Not applicable; PCR-RFLP: polymerase chain reaction-restriction fragment length polymorphism; RT-PCR: Real-time polymerase chain reaction; UK: United Kingdom.

### Overall and stratified analyses

**Figure 2.**
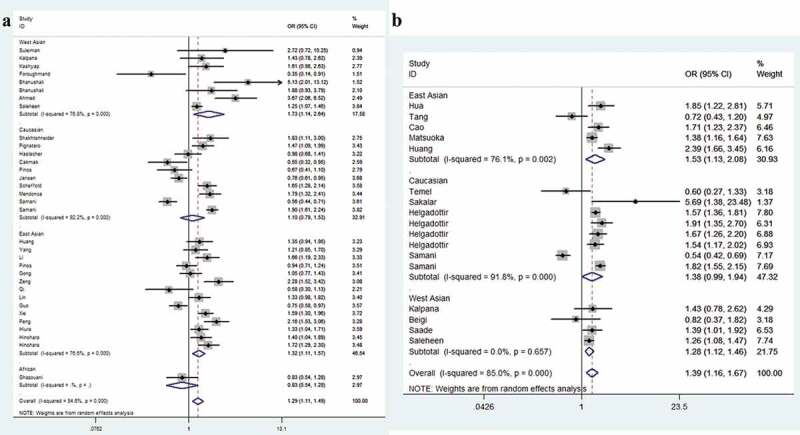
Forest plot of the association between *CDKN2B-AS1* rs1333049 G/C (a), rs4977574 A/G (b) variants and risk of CHD (homozygous contrast, random-effects) in stratified analysis by race.

**Figure 3.**
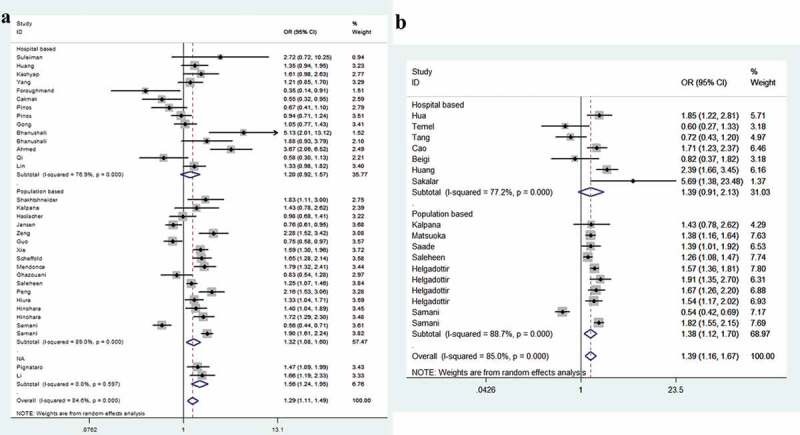
Subgroup analysis by source of control between variation of *CDKN2B-AS1* rs1333049 G/C (a), rs4977574 A/G (b) and risk of CHD (homozygous contrast, random-effects).

**Figure 4.**
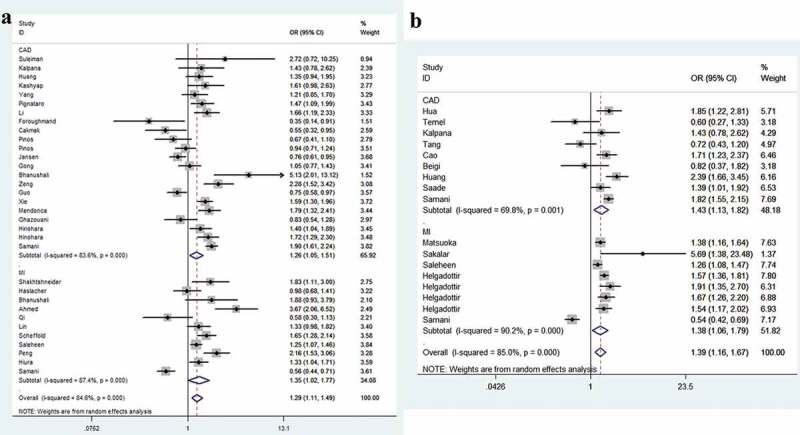
Forest plot of the association between variation of *CDKN2B-AS1* rs1333049 G/C (a), rs4977574 A/G (b) and CHD susceptibility in stratified analysis by phenotype.

The strength of the correlation between *CDKN2B-AS1* SNPs rs1333049 and rs4977574 is summarized in Table 2. For the rs1333049 G/C variation, when all studies pooled together, we observed that individuals carrying CC allele had a 1.29-fold higher risk of CHD than those carrying GG allele (95%CI = 1.11–1.49, *P* = 0.001, Figure 2(a)). In subgroup analyses, we revealed that West Asians with CC allele had a 1.73-fold increased susceptibility than those with GG allele (95%CI = 1.14–2.64, *P* = 0.011). For East Asians, the ratio was 1.32 (95%CI = 1.11–1.57, *P* = 0.001, Figure 2(a)). Moreover, similar findings were indicated for the subgroup with population-based control (C allele vs. G allele, OR = 1.15, 95%CI = 1.04–1.27, *P* = 0.006; CC vs. GG, OR = 1.32, 95%CI = 1.08–1.60, *P* = 0.006; dominant model, OR = 1.17, 95%CI = 1.02–1.35, *P* = 0.028; and recessive model, OR = 1.23, 95%CI = 1.08–1.39, *P* = 0.002, Figure 3(a)). In stratification by phenotype of CHD, we identified that individuals with CC allele had a 1.26-fold higher risk of coronary artery disease than those with GG allele (95%CI = 1.05–1.51, *P* = 0.012). For myocardial infarction groups, the ratio was 1.25 (95%CI = 1.01–1.53, *P* = 0.037, Figure 4(a)). For the rs4977574 A/G variant, a positive association was observed for all studies when combined. Individuals carrying GG allele had a 1.39-fold higher risk of CHD than those carrying AA allele (95%CI = 1.16–1.67, *P* < 0.001, Figure 2(b)). Stratification analysis revealed West Asians with GG allele had a 1.28-fold increased susceptibility than those with AA allele (95%CI = 1.12–1.46, *P* < 0.001, Figure 2(b)). For East Asians the ratio was 1.53 (95%CI = 1.13–2.08, *P* = 0.006, Figure 3(b)). In subgroup analysis by phenotype, we revealed that individuals carrying GG allele had a 1.43-fold increased susceptibility of coronary artery disease than those with AA allele (95%CI = 1.13–1.82, *P* = 0.004). The ratio was 1.38 in myocardial infarction groups (95%CI = 1.06–1.79, *P* = 0.018, Figure 4(b)).

**Table 2.** Stratified analysis of CDKN2B-AS1 rs1333049 and rs4977574 variants on susceptibility to coronary heart disease.

Variables	N^a^	Case/	OR(95%CI) *P*_heter_ *P*	OR(95%CI) *P*_heter_ *P*	OR(95%CI) *P*_heter_ *P*	OR(95%CI) *P*_heter_ *P*	OR(95%CI) *P*_heter_ *P*
		Control	M-allele vs. W-allele	MW vs. WW	MM vs. WW	MM+MW vs. WW	MM vs. MW+WW
rs1333049 G/C							
Total	33	20365/29413	1.13(1.05–1.21) <0.001 0.001	1.08(0.99–1.18) <0.001 0.076	1.29(1.11–1.49) <0.001 0.001	1.14(1.03–1.27) <0.001 0.011	1.21(1.10–1.34) <0.001 < 0.001
Ethnicity							
West Asian	8	3921/4023	1.25(1.07–1.45) 0.005 0.005	1.10(0.98–1.23) 0.072 0.091	1.73(1.14–2.64) <0.001 0.011	1.26(0.98–1.62) 0.018 0.066	1.52(1.14–2.01) 0.002 0.004
Caucasian	14	6979/12696	1.05(0.89–1.24) <0.001 0.575	1.01(0.82–1.25) <0.001 0.916	1.10(0.79–1.53) <0.001 0.565	1.04(0.81–1.33) <0.001 0.743	1.10(0.89–1.35) <0.001 0.397
East Asian	10	9173/12371	1.15(1.06–1.25) <0.001 0.001	1.12(1.02–1.23) 0.039 0.023	1.32(1.11–1.57) <0.001 0.001	1.18(1.05–1.33) <0.001 0.005	1.23(1.09–1.39) <0.001 0.001
African	1	292/323	0.90(0.72–1.25) – 0.381	0.92(0.63–1.34) – 0.661	0.83(0.54–1.28) – 0.395	0.89(0.62–1.26) – 0.501	0.87(0.61–1.25) – 0.465
Source							
HB	14	4510/5840	1.07(0.96–1.20) <0.001 0.229	1.03(0.88–1.21) 0.008 0.696	1.20(0.92–1.57) <0.001 0.178	1.07(0.90–1.27) <0.001 0.437	1.15(0.95–1.39) <0.001 0.144
PB	17	14589/22338	1.15(1.04–1.27) <0.001 0.006	1.11(0.98–1.25) <0.001 0.092	1.32(1.08–1.60) <0.001 0.006	1.17(1.02–1.35) <0.001 0.028	1.23(1.08–1.39) <0.001 0.002
NA	2	1266/1235	1.27(1.13–1.42) 0.475 < 0.001	1.13(0.92–1.39) 0.810 0.249	1.56(1.24–1.95) 0.597 < 0.001	1.29(1.06–1.56) 0.667 0.012	1.43(1.20–1.69) 0.585 < 0.001
Phenotype							
CAD	22	13262/16209	1.12(1.02–1.22) <0.001 0.013	1.06(0.94–1.20) <0.001 0.307	1.26(1.05–1.51) <0.001 0.012	1.12(0.98–1.29) <0.001 0.092	1.20(1.08–1.34) <0.001 0.001
MI	11	7103/13204	1.15(1.01–1.30) <0.001 0.034	1.11(0.98–1.27) 0.010 0.102	1.35(1.02–1.77) <0.001 0.033	1.17(1.00–1.38) <0.001 0.055	1.25(1.01–1.53) <0.001 0.037
rs4977574 A/G							
Total	17	15550/19460	1.18(1.07–1.29) <0.001 0.001	1.16(1.05–1.29) 0.001 0.003	1.39(1.16–1.67) <0.001 < 0.001	1.24(1.09–1.40) <0.001 0.001	1.26(1.10–1.44) <0.001 0.001
Ethnicity							
West Asian	4	4294/3528	1.13(1.06–1.21) 0.678 < 0.001	1.03(0.92–1.16) 0.372 0.607	1.28(1.12–1.46) 0.657 < 0.001	1.11(0.99–1.25) 0.507 0.062	1.24(1.12–1.38) 0.445 < 0.001
Caucasian	8	7392/12030	1.18(1.00–1.40) <0.001 0.055	1.18(0.99–1.40) <0.001 0.071	1.33(0.99–1.94) <0.001 0.061	1.25(0.99–1.56) <0.001 0.057	1.23(0.97–1.56) <0.001 0.083
East Asian	5	3864/3902	1.21(1.03–1.43) <0.001 0.023	1.22(1.10–1.37) 0.633 < 0.001	1.53(1.13–2.08) 0.002 0.006	1.31(1.18–1.45) 0.137 < 0.001	1.29(0.97–1.72) <0.001 0.080
Source							
HB	7	2257/1892	1.17(0.93–1.47) <0.001 0.178	1.27(1.08–1.48) 0.190 0.003	1.39(0.91–2.13) <0.001 0.124	1.27(0.96–1.66) 0.012 0.093	1.23(0.87–1.74) <0.001 0.242
PB	10	13293/17568	1.18(1.06–1.31) <0.001 0.002	1.14(1.02–1.28) <0.001 0.027	1.38(1.12–1.70) <0.001 0.003	1.22(1.05–1.40) <0.001 0.007	1.27(1.09–1.47) <0.001 0.002
Phenotype							
CAD	9	5747/5660	1.18(1.04–1.34) <0.001 0.013	1.28(1.16–1.41) 0.199 < 0.001	1.43(1.13–1.82) 0.001 0.004	1.28(1.09–1.51) 0.031 0.003	1.28(1.04–1.57) <0.001 0.017
MI	8	9803/13800	1.18(1.03–1.35) <0.001 0.016	1.14(0.99–1.31) 0.001 0.065	1.38(1.06–1.79) <0.001 0.018	1.22(1.03–1.45) <0.001 0.025	1.27(1.02–1.50) <0.001 0.028

CAD: Coronary artery disease; HB: Hospital based; MI: Myocardial Infarction; NA: Not applicable; PB: Population based.

^a^Number of comparisons

*P*_heter_: *P* value of *Q*-test for heterogeneity test.

### In silico analysis of CDKN2B

**Figure 5.**
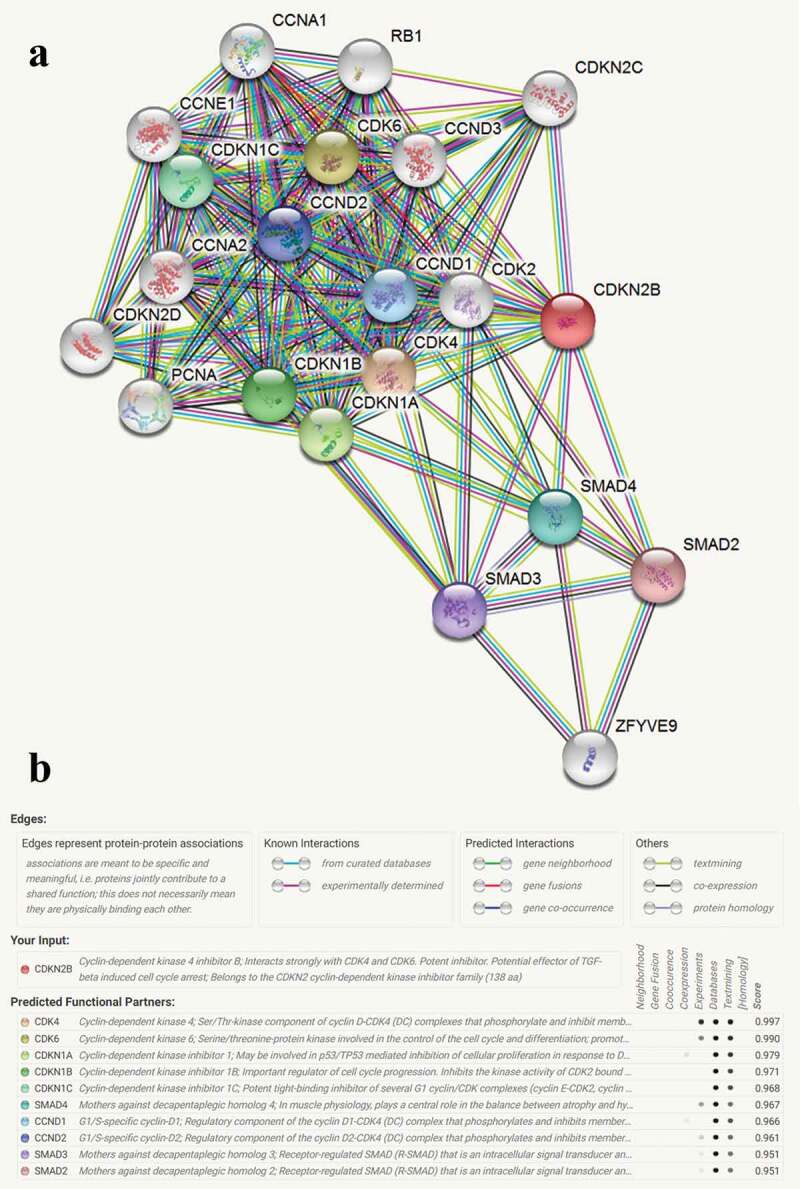
Protein-protein crosstalk of CDKN2B (a). The top 10 most relevant feature partners are as follows: Cyclin-dependent kinase (CDK) 4, CDK 6, Cyclin-dependent kinase inhibitor (CDKN) 1A, CDKN 1B, CDKN 1 C, Mothers against decapentaplegic homolog (SMAD) 4, G1/S-specific cyclin-D (CCND) 1, CCND 2, SMAD 3, SMAD 2 (b).

**Figure 6.**
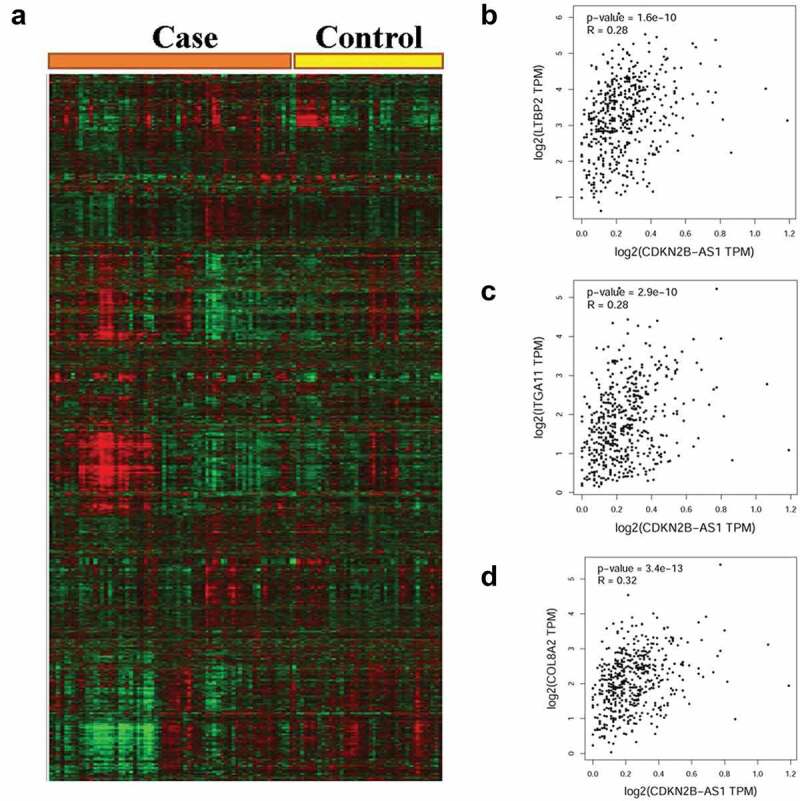
Differentially expressed genes between CHD and control group (a). The probably correlated gene with CDKN2B includes the latent transforming factor beta binding protein (LTBP) 2, (b), integrin subunit alpha 11 (ITGA11, c), collagen type VIII alpha 2 chain (COL8A2, d).

Protein-protein crosstalk of CDKN2B was investigated by the STRING tools. Interaction of least 20 proteins with CDKN2B was identified in Figure 5. The most relevant interactions were with the following proteins: Cyclin-Dependent Kinase (CDK) 4, CDK 6, Cyclin-Dependent Kinase Inhibitor (CDKN) 1A, CDKN 1B, CDKN 1 C, Mothers against decapentaplegic homolog (SMAD) 4, G1/S-specific cyclin-D (CCND) 1, CCND 2, SMAD 3, and SMAD 2 (Figure 5(b)). The online database was also utilized to assess the differentially expressed genes between the CHD and control groups (Figure 6(a)). The most probable correlations with CDKN2B in CHD included the genes for latent transforming factor beta binding protein 2 (LTBP2, Figure 6(b)), integrin subunit alpha 11 (ITGA11, Figure 6(c)), and collagen type VIII alpha 2 chain (COL8A2, Figure 6(d)).

### Publication bias and sensitivity analysis

**Figure 7.**
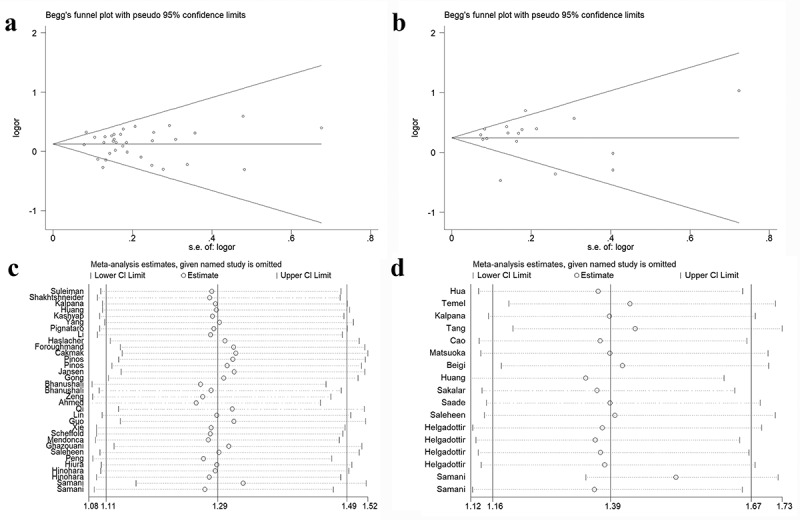
Publication bias and sensitivity analysis for *CDKN2B-AS1* rs1333049 G/C and rs4977574 A/G polymorphisms. We revealed no evidence of publication bias according to rs1333049 G/C (a) and rs4977574 (b). No significant change of the result was detected in the sensitivity analysis for rs1333049 G/C (c) and rs4977574 (d) variants.

We constructed the Begg’s funnel plots to detect the publication bias among the included studies. We identified no significant asymmetry of the funnel plots in any of these models when evaluating the variants of rs1333049 (Figure 7(a), *P* > 0.05) and rs4977574 (Figure 7(b), *P* > 0.05). Furthermore, we conducted sensitivity analysis by removing single studies. Single study did not have an impact on the significance of ORs for both rs1333049 G/C (Figure 7(c)) and rs4977574 A/G (Figure 7(d)) polymorphisms.

## Discussion

CHD is still the main cause of mortality globally and imposes a huge social and economic burden [59,60]. The relationship between the *CDKN2B-AS1* variants rs1333049 and rs4977574 and the risk of CHD has been previously reported; however, a comprehensive analysis of the relationship was not available. Several meta-analyses have pooled the data of various studies; however, the number of studies included was insufficient. In 2018, Xu *et al* evaluated six articles on *CDKN2B-AS1* SNP rs4977574 indicating increased likelihood of CHD due to the variation [19]. Hu *et al* in 2019 evaluated the association between SNP rs1333049 and CHD using 7 studies and reported increased risk of CHD with rs1333049 in the East Asian population [61]. The present analysis, which involved a total of 50 case-control studies with 35,915 CHD patients and 48,873 control subjects, is by far the most comprehensive analysis evaluating the relationship between *CDKN2B-AS1* variants rs1333049 and rs4977574 and the risk of CHD. Our analysis revealed a significant association of rs1333049 G/C and rs4977574 A/G variants with the likelihood of CHD, when all studies were pooled together.

For the SNP rs1333049, C allele was a risk factor for both West Asians and East Asians in the subgroup analysis by race. In the stratified analysis by source of control population, there is a positive correlation between rs1333049 variant and population-based studies. In a subgroup analysis based on disease type, we observed that individuals carrying CC allele had an increased susceptibility of coronary artery disease and myocardial infarction patients’ group. Our conclusion is not consistent with the meta-analysis performed by Xie et al, who observed no positive relationship between this variant and susceptibility of myocardial infarction groups (allele contrast, *P* value = 0.17, OR = 0.87, 95% confidence intervals = 0.72–1.06; dominant comparison, *P* value = 0.14, OR = 0.83, 95% confidence intervals = 0.64–1.07; recessive genetic model, *P* value = 0.28, OR = 1.25, 95% confidence intervals = 0.84–1.86) [18]. A possible reason for the difference in study outcomes may be the relatively small number of studies included in their meta-analysis. For the SNP rs4977574, we detected a significant correlation between the G allele and the risk of CHD among West Asian and East Asian populations in a stratification analysis by ethnicity and the findings are consistent with the results in a previous study [62]. In stratification analysis by control population source, there was a positive correlation with population-based studies. Based on previous randomized controlled trial, *CDKN2B-AS1* rs1333049 G/C and rs4977574 A/G variants were not correlated with higher risk in African patients with CHD [63]. Evidence from genome-wide association study showed that no major locus could individually reveal the high risk of coronary heart disease in African Americans [64]. Moreover, we checked the MAFs in worldwide populations based on the online database. The MAF for the *CDKN2B-AS1* rs1333049 G/C variant in Africans is 0.21. It is lower than that in other populations and global average. Similar result was indicated for the rs4977574 A/G variant. A possible reason is that *CDKN2B-AS1* rs1333049 G/C and rs4977574 A/G variants may be not associated with the CHD susceptibility in African population. Additionally, an online database was employed to explore differentially expressed genes between the CHD and control groups. We found that expression of *LTBP2, ITGA11*, and *COL8A2* correlated with the expression of CDKN2B in CHD. The online database contains scant data on the specific mechanism of these genes. Future functional analyses and *in vitro* experiments are needed to demonstrate the correlations in detail.

The current analysis has several limitations. First, we observed significant heterogeneity in the overall analysis when evaluating the *CDKN2B-AS1* rs1333049 G/C and rs4977574 A/G variations. Although the DerSimonian and Laird method was employed [65], potential bias may influence the conclusion. Second, the pathogenesis of CHD is very complex. Thus, a single gene polymorphism is unlikely to make a significant contribution to its development. All OR values obtained in the current study are all < 2. Therefore, further studies elucidating the gene-gene or gene-environment connections to demonstrate correlation are recommended. In addition, the analysis of the protein-protein crosstalk of CDKN2B by the STRING tool, identified interactions with more than 20 proteins (Figure 5), however, these interactions need be confirmed by *in vitro* and *in vivo* analyses. Third, the study does not include adjusted analysis for sex, lifestyle, and smoking exposure, which may have helped in better segregation and evaluation of the different groups.

## Conclusion

Taken together, our study demonstrates that *CDKN2B-AS1* rs1333049 C allele and rs4977574 G allele is correlated with the risk of CHD. These polymorphisms may serve as genetic biomarkers for CHD, especially in people of East and West Asian ancestry.

## Data Availability

All data in the present research are available from this manuscript.

## References

[1] Global Burden of Disease Study 2013 Collaborators. Global, regional, and national incidence, prevalence, and years lived with disability for 301 acute and chronic diseases and injuries in 188 countries, 1990-2013: a systematic analysis for the Global Burden of Disease Study 2013. Lancet. 2015;386(9995):743–800. 26063472 10.1016/S0140-6736(15)60692-4 PMC4561509

[2] Girelli D, Martinelli N, Peyvandi F, et al. Genetic architecture of coronary artery disease in the genome-wide era: implications for the emerging “golden dozen” loci. Semin Thromb Hemost. 2009;35(7):671–682. 20013534 10.1055/s-0029-1242721

[3] McPherson R, Pertsemlidis A, Kavaslar N, et al. A common allele on chromosome 9 associated with coronary heart disease. Science. 2007;316(5830):1488–1491. 17478681 10.1126/science.1142447 PMC2711874

[4] Willer CJ, Sanna S, Jackson AU, et al. Newly identified loci that influence lipid concentrations and risk of coronary artery disease. Nat Genet. 2008;40(2):161–169. 18193043 10.1038/ng.76 PMC5206900

[5] Jarinova O, Stewart AF, Roberts R, et al. Functional analysis of the chromosome 9p21.3 coronary artery disease risk locus. Arterioscler Thromb Vasc Biol. 2009;29(10):1671–1677. 19592466 10.1161/ATVBAHA.109.189522

[6] Congrains A, Kamide K, Katsuya T, et al. CVD-associated non-coding RNA, ANRIL, modulates expression of atherogenic pathways in VSMC. Biochem Biophys Res Commun. 2012;419(4):612–616. 22382030 10.1016/j.bbrc.2012.02.050

[7] Congrains A, Kamide K, Oguro R, et al. Genetic variants at the 9p21 locus contribute to atherosclerosis through modulation of ANRIL and CDKN2A/B. Atherosclerosis. 2012;220(2):449–455. 22178423 10.1016/j.atherosclerosis.2011.11.017

[8] Cunnington MS, Keavney B. Genetic mechanisms mediating atherosclerosis susceptibility at the chromosome 9p21 locus. Curr Atheroscler Rep. 2011;13(3):193–201. 21487702 10.1007/s11883-011-0178-z

[9] Ferreira LE, Secolin R, Lopes-Cendes I, et al. Association and interaction of genetic variants with occurrence of ischemic stroke among Brazilian patients. Gene. 2019;695:84–91. 30738964 10.1016/j.gene.2019.01.041

[10] Restrepo NA, Laper SM, Farber-Eger E, et al. Local genetic ancestry in CDKN2B-AS1 is associated with primary open-angle glaucoma in an African American cohort extracted from de-identified electronic health records. BMC Med Genomics. 2018;11(Suppl 3):70. 30255811 10.1186/s12920-018-0392-4 PMC6157155

[11] Hsu A, Dalbeth N, Gow P, et al. No evidence for association of Chr 9p21 variant rs1333049 with gout in New Zealand case-control sample sets. Rheumatology (Oxford). 2012;51(6):1129–1130. 22396608 10.1093/rheumatology/kes029 PMC3465700

[12] Gong WJ, Peng JB, Yin JY, et al. Association between well-characterized lung cancer lncRNA polymorphisms and platinum-based chemotherapy toxicity in Chinese patients with lung cancer. Acta Pharmacol Sin. 2017;38(4):581–590. 28260796 10.1038/aps.2016.164 PMC5386317

[13] Broadbent HM, Peden JF, Lorkowski S, et al. Susceptibility to coronary artery disease and diabetes is encoded by distinct, tightly linked SNPs in the ANRIL locus on chromosome 9p. Hum Mol Genet. 2008;17(6):806–814. 18048406 10.1093/hmg/ddm352

[14] Krimpenfort P, Ijpenberg A, Song ZY, et al. p15Ink4b is a critical tumour suppressor in the absence of p16Ink4a. Nature. 2007;448(7156):943–946. 17713536 10.1038/nature06084

[15] Helgadottir A, Thorleifsson G, Manolescu A, et al. A common variant on chromosome 9p21 affects the risk of myocardial infarction. Science. 2007;316(5830):1491–1493. 17478679 10.1126/science.1142842

[16] Gioli-Pereira L, Santos PC, Ferreira NE, et al. Higher incidence of death in multi-vessel coronary artery disease patients associated with polymorphisms in chromosome 9p21. BMC Cardiovasc Disord. 2012;12:61. 22856518 10.1186/1471-2261-12-61 PMC3469382

[17] Correia M, Branco CC, Bruffaerts N, et al. Genetic risk assessment for cardiovascular disease in Azoreans (Portugal): a general population-based study. Gene. 2013;532(1):132–139. 24042129 10.1016/j.gene.2013.08.099

[18] Xie Y, Zhao D, Dong P, et al. Effects of ANRIL polymorphisms on the likelihood of coronary artery disease: a meta-analysis. J Cell Biochem. 2018;120(4):6113–6119. 30387168 10.1002/jcb.27898

[19] Xu B, Fang Z, He S, et al. ANRIL polymorphism rs4977574 is associated with increased risk of coronary artery disease in Asian populations: A meta-analysis of 12,005 subjects. Medicine (Baltimore). 2018;97(39):e12641. 30278588 10.1097/MD.0000000000012641 PMC6181537

[20] Helgadottir A, Thorleifsson G, Magnusson KP, et al. The same sequence variant on 9p21 associates with myocardial infarction, abdominal aortic aneurysm and intracranial aneurysm. Nat Genet. 2008;40(2):217–224. 18176561 10.1038/ng.72

[21] Samani NJ, Erdmann J, Hall AS, et al. Genomewide association analysis of coronary artery disease. N Engl J Med. 2007;357(5):443–453. 17634449 10.1056/NEJMoa072366 PMC2719290

[22] Hiura Y, Fukushima Y, Yuno M, et al. Validation of the association of genetic variants on chromosome 9p21 and 1q41 with myocardial infarction in a Japanese population. Circ J. 2008;72(8):1213–1217. 18654002 10.1253/circj.72.1213

[23] Peng WH, Lu L, Zhang Q, et al. Chromosome 9p21 polymorphism is associated with myocardial infarction but not with clinical outcome in Han Chinese. Clin Chem Lab Med. 2009;47(8):917–922. 19548844 10.1515/CCLM.2009.215

[24] Ghazouani L, Khalifa SB, Abboud N, et al. Association of three polymorphisms selected from a genome-wide association study with coronary heart disease in the Tunisian population. J Thromb Thrombolysis. 2010;29(1):114–118. 19373437 10.1007/s11239-009-0336-0

[25] Saleheen D, Alexander M, Rasheed A, et al. Association of the 9p21.3 locus with risk of first-ever myocardial infarction in Pakistanis: case-control study in South Asia and updated meta-analysis of Europeans. Arterioscler Thromb Vasc Biol. 2010;30(7):1467–1473. 20395598 10.1161/ATVBAHA.109.197210

[26] Guo J, Feng Y, Li H, et al. Association study of single-nucleotide polymorphisms on chromosome 1p13, 1p32, 9p21 and 19p13 with cardiovascular diseases in Chinese Han population: a case-control study. J Hypertens. 2016;5:4.

[27] Lin HF, Tsai PC, Liao YC, et al. Chromosome 9p21 genetic variants are associated with myocardial infarction but not with ischemic stroke in a Taiwanese population. J Investig Med. 2011;59(6):926–930. 10.2310/JIM.0b013e318214ea49 21415773

[28] Mendonça I, Dos Reis RP, Pereira A, et al. Independent association of the variant rs1333049 at the 9p21 locus and coronary heart disease. Rev Port Cardiol. 2011;30(6):575–591. 21874923

[29] Saade S, Cazier JB, Ghassibe-Sabbagh M, et al. Large scale association analysis identifies three susceptibility loci for coronary artery disease. PLoS One. 2011;6(12):e29427. 22216278 10.1371/journal.pone.0029427 PMC3246490

[30] Scheffold T, Kullmann S, Huge A, et al. Six sequence variants on chromosome 9p21.3 are associated with a positive family history of myocardial infarction: a multicenter registry. BMC Cardiovasc Disord. 2011;11:9. 21385355 10.1186/1471-2261-11-9 PMC3061953

[31] Xie F, Chu X, Wu H, et al. Replication of putative susceptibility loci from genome-wide association studies associated with coronary atherosclerosis in Chinese Han population. PLoS One. 2011;6(6):e20833. 21698238 10.1371/journal.pone.0020833 PMC3116833

[32] Zeng Q, Yuan Y, Wang S, et al. Polymorphisms on chromosome 9p21 confer a risk for acute coronary syndrome in a Chinese Han population. Can J Cardiol. 2013;29(8):940–944. 23454037 10.1016/j.cjca.2012.11.028

[33] Qi L, Li JM, Sun H, et al. Association between gene polymorphisms and myocardial infarction in Han Chinese of Yunnan province. Zhonghua Yi Xue Yi Chuan Xue Za Zhi. 2012;29(4):413–419. 22875497 10.3760/cma.j.issn.1003-9406.2012.04.008

[34] Ahmed W, Ali IS, Riaz M, et al. Association of ANRIL polymorphism (rs1333049: C>G)with myocardial infarction and its pharmacogenomic role in hypercholesterolemia. Gene. 2013;515(2):416–420. 23266621 10.1016/j.gene.2012.12.044

[35] Bhanushali AA, Contractor A, Das BR. Variant at 9p21 rs1333049 is associated with age of onset of coronary artery disease in a Western Indian population: a case control association study. Genet Res (Camb). 2013;95(5):138–145. 24246088 10.1017/S0016672313000189

[36] Sakalar C, Gurbuz E, Kalay N, et al. Higher frequency of rs4977574 (the G Allele) on chromosome 9p21.3 in patients with myocardial infarction as revealed by PCR-RFLP analysis. Tohoku J Exp Med. 2013;230(3):171–176. 23856978 10.1620/tjem.230.171

[37] Gong L, Chen J, Lu J, et al. The 9p21 locus is associated with coronary artery disease and cardiovascular events in the presence (but not in the absence) of coronary calcification. PLoS One. 2014;9(4):e94823. 24732910 10.1371/journal.pone.0094823 PMC3986239

[38] Huang Y, Ye H, Hong Q, et al. Association of CDKN2BAS polymorphism rs4977574 with coronary heart disease: a case-control study and a meta-analysis. Int J Mol Sci. 2014;15(10):17478–17492. 25268619 10.3390/ijms151017478 PMC4227174

[39] Jansen MD, Knudsen GP, Myhre R, et al. Genetic variants in loci 1p13 and 9p21 and fatal coronary heart disease in a Norwegian case-cohort study. Mol Biol Rep. 2014;41(5):2733–2743. 24728607 10.1007/s11033-014-3096-7

[40] Pinós T, Fuku N, Cámara Y, et al. The rs1333049 polymorphism on locus 9p21.3 and extreme longevity in Spanish and Japanese cohorts. Age (Dordr). 2014;36(2):933–943. 24163049 10.1007/s11357-013-9593-0 PMC4039251

[41] Beigi S, Ghaderian S, Doosti A. Investigation of the association between rs4977574 A>G Polymorphism in ANRIL gene and coronary artery disease in Iranian population. Int J Cardiovasc Res. 2015;9(3):139–144.

[42] Çakmak HA, Bayoğlu B, Durmaz E, et al. Evaluation of association between common genetic variants on chromosome 9p21 and coronary artery disease in Turkish population. Anatol J Cardiol. 2015;15(3):196–203. 25333979 10.5152/akd.2014.5285 PMC5337054

[43] Foroughmand AM, Nikkhah E, Galehdari H, et al. Association study between coronary artery disease and rs1333049 and rs10757274 polymorphisms at 9p21 locus in South-West Iran. Cell J. 2015;17(1):89–98. 25870838 10.22074/cellj.2015.515 PMC4393676

[44] Matsuoka R, Abe S, Tokoro F, et al. Association of six genetic variants with myocardial infarction. Int J Mol Med. 2015;35(5):1451–1459. 25738804 10.3892/ijmm.2015.2115

[45] Cao XL, Yin RX, Huang F, et al. Chromosome 9p21 and ABCA1 genetic variants and their interactions on coronary heart disease and ischemic stroke in a Chinese Han population. Int J Mol Sci. 2016;17(4):586. 27096864 10.3390/ijms17040586 PMC4849041

[46] Haslacher H, Perkmann T, Ratzinger F, et al. 9p21.3 risk locus is associated with first-ever myocardial infarction in an Austrian cohort. J Cardiovasc Med (Hagerstown). 2016;17(8):595–600. 25032714 10.2459/JCM.0000000000000183

[47] Li Q, Peng W, Li H, et al. Association of the single nucleotide polymorphism in chromosome 9p21 and chromosome 9q33 with coronary artery disease in Chinese population. BMC Cardiovasc Disord. 2017;17(1):255. 28962556 10.1186/s12872-017-0685-0 PMC5622451

[48] Pignataro P, Pezone L, Di Gioia G, et al. Association study between coronary artery disease and rs1333049 polymorphism at 9p21.3 locus in Italian population. J Cardiovasc Transl Res. 2017;10(5–6):455–458. 28639227 10.1007/s12265-017-9758-9

[49] Tang O, Lv J, Cheng Y, et al. The correlation between 9p21 chromosome rs4977574 polymorphism genotypes and the development of coronary artery heart disease. Cardiovasc Toxicol. 2017;17(2):185–189. 27240780 10.1007/s12012-016-9372-0

[50] Kashyap S, Kumar S, Agarwal V, et al. The association of polymorphic variants, rs2267788, rs1333049 and rs2383207 with coronary artery disease, its severity and presentation in North Indian population. Gene. 2018;648:89–96. 29309886 10.1016/j.gene.2018.01.021

[51] Yang J, Gu L, Guo X, et al. LncRNA ANRIL Expression and ANRIL gene polymorphisms contribute to the risk of ischemic stroke in the Chinese Han population. Cell Mol Neurobiol. 2018;38(6):1253–1269. 29881905 10.1007/s10571-018-0593-6 PMC11481959

[52] Huang K, Zhong J, Li Q, et al. Effects of CDKN2B-AS1 polymorphisms on the susceptibility to coronary heart disease. Mol Genet Genomic Med. 2019;7(11):e955. 31496134 10.1002/mgg3.955 PMC6825846

[53] Kalpana B, Murthy DK, Balakrishna N, et al. Genetic variants of chromosome 9p21.3 region associated with coronary artery disease and premature coronary artery disease in an Asian Indian population. Indian Heart J. 2019;71(3):263–271. 31543200 10.1016/j.ihj.2019.04.005 PMC6796635

[54] Shakhtshneider E, Orlov P, Semaev S, et al. Analysis of polymorphism rs1333049 (Located at 9P21.3) in the white population of Western Siberia and associations with clinical and biochemical markers. Biomolecules. 2019;9(7):pii: E290. 10.3390/biom9070290 PMC6681349 31330999

[55] Suleiman AA, Muhsin H, Abdulkareem RA, et al. Association study of two single nucleotide polymorphisms rs10757278 and rs1333049 with atherosclerosis, a case-control study from Iraq. Mol Biol Res Commun. 2019;8(3):99–102. 31998810 10.22099/mbrc.2019.33818.1406 PMC6802693

[56] Temel ŞG, Ergören MÇ. The association between the chromosome 9p21 CDKN2B-AS1 gene variants and the lipid metabolism: A pre-diagnostic biomarker for coronary artery disease. Anatol J Cardiol. 2019;21(1):31–38. 30587704 10.14744/AnatolJCardiol.2018.90907 PMC6382903

[57] Hua L, Yuan JX, He S, et al. Analysis on the polymorphisms of site RS4977574, and RS1333045 in region 9p21 and the susceptibility of coronary heart disease in Chinese population. BMC Med Genet. 2020;21(1):36. 32066403 10.1186/s12881-020-0965-x PMC7026955

[58] Hinohara K, Nakajima T, Takahashi M, et al. Replication of the association between a chromosome 9p21 polymorphism and coronary artery disease in Japanese and Korean populations. J Hum Genet. 2008;53(4):357–359. 18264662 10.1007/s10038-008-0248-4

[59] Owan TE, Roe MT, Messenger JC, et al. Contemporary use of adjunctive thrombectomy during primary percutaneous coronary intervention for ST-elevation myocardial infarction in the United States. Catheter Cardiovasc Interv. 2012;80:1173–1180. 22511575 10.1002/ccd.24306

[60] Chien KL, Hsu HC, Su TC, et al. Constructing a point-based prediction model for the risk of coronary artery disease in a Chinese community: a report from a cohort study in Taiwan. Int J Cardiol. 2012;157(2):263–268. 22503568 10.1016/j.ijcard.2012.03.017

[61] Hu L, Su G, Wang X. The roles of ANRIL polymorphisms in coronary artery disease: a meta-analysis. Biosci Rep. 2019;39(12):pii: BSR20181559. 10.1042/BSR20181559 PMC6923330 30814313

[62] Guo J, Li W, Wu Z, et al. Association between 9p21.3 genomic markers and coronary artery disease in East Asians: a meta-analysis involving 9,813 cases and 10,710 controls. Mol Biol Rep. 2013;40(1):337–343. 23086272 10.1007/s11033-012-2066-1

[63] Gong Y, Beitelshees AL, Cooper-DeHoff RM, et al. Chromosome 9p21 haplotypes and prognosis in white and black patients with coronary artery disease. Circ Cardiovasc Genet. 2011;4(2):169–178. 21372283 10.1161/CIRCGENETICS.110.959296 PMC3101633

[64] Lettre G, Palmer CD, Young T, et al. Genome-wide association study of coronary heart disease and its risk factors in 8,090 African Americans: the NHLBI CARe project. PLoS Genet. 2011;7(2):e1001300. 21347282 10.1371/journal.pgen.1001300 PMC3037413

[65] DerSimonian R, Laird N. Meta-analysis in clinical trials. Control Clin Trials. 1986;7(3):177–188. 3802833 10.1016/0197-2456(86)90046-2

